# A Freedom of *Coxiella burnetii* Infection Survey in European Bison (*Bison bonasus*) in Poland

**DOI:** 10.3390/ani11030651

**Published:** 2021-03-01

**Authors:** Michał K. Krzysiak, Martyna Puchalska, Wanda Olech, Krzysztof Anusz

**Affiliations:** 1 Białowieża National Park, Park Pałacowy 11, 17-230 Białowieża, Poland; 2 Institute of Forest Sciences, Faculty of Civil Engineering and Environmental Sciences, Białystok University of Technology, Wiejska 45 E, 15-351 Białystok, Poland; 3 Department of Food Hygiene and Public Health Protection, Institute of Veterinary Medicine, Warsaw University of Life Sciences—SGGW, Nowoursynowska 159, 02-776 Warsaw, Poland; martyna_puchalska@sggw.edu.pl (M.P.); krzysztof_anusz@sggw.edu.pl (K.A.); 4 Department of Animal Genetics and Conservation, Institute of Animal Science, Warsaw University of Life Sciences—SGGW, Ciszewskiego 8, 02-786 Warsaw, Poland; wanda_olech@sggw.edu.pl

**Keywords:** *Coxiella burnetii*, Q fever, serology, epidemiology, wildlife, European bison

## Abstract

**Simple Summary:**

Q fever is one of the important diseases transmissible from animals to humans. The source of infection can be numerous species of animals including mammals, birds, reptiles, amphibians as well as ticks. The role of wildlife in its epidemiology is poorly understood. Therefore, we examined 523 sera samples obtained from European bison for the presence of specific antibodies to assess whether infection occurs in this species and whether European bison may be an important source of infection in the natural environment as suggested by historical reports. The antibodies were found only in one free-living bull, while two other samples were doubtful. The results suggest the transmission of infection to the European bison was rather accidental and its role as an important source of infection nowadays is unlikely.

**Abstract:**

Q fever is an important zoonosis caused by the intracellular Gram-negative bacteria *Coxiella burnetii*. The source of infection are numerous species of mammals, birds, reptiles and amphibians, as well as ticks. The disease is widespread throughout Europe, but the role of wildlife in its epidemiology is poorly understood. The European bison (*Bison bonasus*) population has been growing European-wide quite dynamically over the last few years. The aim of this study was to determine whether *C. burnetii* infection occurs in European bison and whether it can be considered an important bacterial reservoir in the natural environment. Five hundred and twenty three samples of European bison sera originating from 14 (out of the 26 existing) Polish populations were examined for the presence of specific antibodies using an ID Screen Q Fever Indirect Multi-species ELISA test. Only one (0.19%) serum sample was positive in ELISA, and two other samples were doubtful. The only seropositive animal found in this study was a free-living bull. It suggests possible transmission from domestic cattle by sharing pastures. The transmission of *C. burnetii* into the European bison was rather accidental in the country and its role as an important wild reservoir is unlikely. Since no tests are available for wildlife ruminants there is a need for the adaptation of the available tests.

## 1. Introduction

Q fever is a widely distributed reproductive disease of ruminants caused by intracellular bacteria *Coxiella burnetii*. The pathogen is commonly transmitted to humans and remains an emerging concern for public health. The source of infection are numerous species of mammals, birds, reptiles and amphibians. Ticks are important vectors of *C. burnetii* transmission; therefore, the climate changes observed in the recent decades may provoke the shift of those arthropods into new locations, increasing the risk of tick-borne infections to occur [1]. In humans, infection as a result of a tick bite is rare, while potential sources of infection may be dusty feces of infected ticks, or exposure to dust with dried secretions of the reproductive system spread by the wind up to a distance of 2 km. Free-living animals may be a reservoir and source of *C. burnetii* infection for both humans and domestic animals. Q fever is widely spread all over Europe, however the role of wildlife in its transmission is still poorly understood. Dairy cattle are considered the main reservoir in Poland with the animal-level and herd-level seroprevalence around 25% [2]. *C. burnetii* DNA was detected in 3% of wildlife tested in a survey in the north-west of the country, which included roe deer, red deer and wild boar [3]. In the same study, the presence of bacteria was also confirmed in the ticks *Ixodes ricinus* from the same area. Other reports also confirmed the infection in wild ruminants, rabbits, rodents, foxes and ticks associated with their sylvatic ecosystems [4,5]. Moreover, specific antibodies were found in wild ranging cervids and mouflon [6,7].

European bison are the largest herbivores in Europe. Their conservation strategies should also include the monitoring of zoonotic diseases as an element of the One Health concept. However, Q fever studies have been neglected for the last two decades. The first report on the occurrence of *C. burnetii* in European bison dates back to the nineteen-eighties, when the exposure of forty-seven free-ranging European bison was investigated from Borecka forest (54°5′18.09″ N 21°55′21.467″ E) in connection to a Q fever epidemic in local cattle herds. The presence of specific antibodies was then confirmed in 76% of animals by the microagglutination test and complement fixation test (CFT) [8]. Furthermore, earlier in 1980–1983, a natural foci of Q fever could have been located in the Białowieska forest (52°42′9.861″ N 23°51′4.52″ E) [9], where the conservation of the endangered species after their complete extinction from the wild was initiated. Additionally, transmission to humans was suspected as 10% of European bison caretakers from Białowieża National Park (52°42′9.861″ N 23°51′4.52″ E) became infected [8]. Further studies excluded exposure of European bison to *C. burnetii* by serological testing in 122 European bison from Białowieska forest in years 1991–2001 [10,11]. The population of the species, which remains listed by the International Union for Conservation (IUCN) of Nature’s Red List of Threatened Species increases successfully thanks to many national and international projects, reaching already \ total of over 8400 individuals. They are most often reared in the enclosures but wild European bison population with frequent contact with people and domestic animals significantly increases [12]. In 2019, 74% of European bison were free-living [12].

Taking into account abovementioned, the aim of this study was to determine whether *C. burnetii* infection occurs in the European bison and whether it can be considered an important bacterial reservoir in the natural environment.

## 2. Materials and Methods

### 2.1. Study Design

To calculate the representative number of European bison in each of the populations to substantiate a prevalence of 1.5% or below with the overall α error set to 0.05, the FreeCalc software of Epitools [13,14] was used. The mean population size was determined by European bison Pedigree Books (2013–2018). The targeted population size number increased from 1196 animals in 2013 to 1478 in 2018 with the mean value of 1377 individuals [12]. Considering the design prevalence at 1%, test sensitivity and specificity of about 100% and desired type I and II errors at 0.05, the targeted sample size for the whole population tested was 267. However, for the smaller herds, all the animals or all the available samples were included in the study.

The samples were collected during the monitoring of European bison health in the years 2011–2017 as a part of the scientific cooperation of National Veterinary Research Institute in Puławy (NVRI) with herd/population managers based on: the opinion of the Minister of the Environment of 27 October 2014 (ZOP/06-061/51/2014); the Decision of the General Director for Environmental Protection of 31 December 2014 (DZP-WG.6401.06.23.2014.km.2); individual permits of the Director of the Białowieża National Park of 20 December 2013 (PN/061/22/2013) and 15 May 2017 (PN/061/14/2017) and permits of the Kobiór Forest District (ZG-7326 (1) -9/2014). Since 2017, the sample collection has been carried out as part of the project “Complex project of European bison conservation by State Forests” financed by the Forest Fund (Poland) in accordance with contract No. OR.271.3.10.2017.

**Figure 1:**
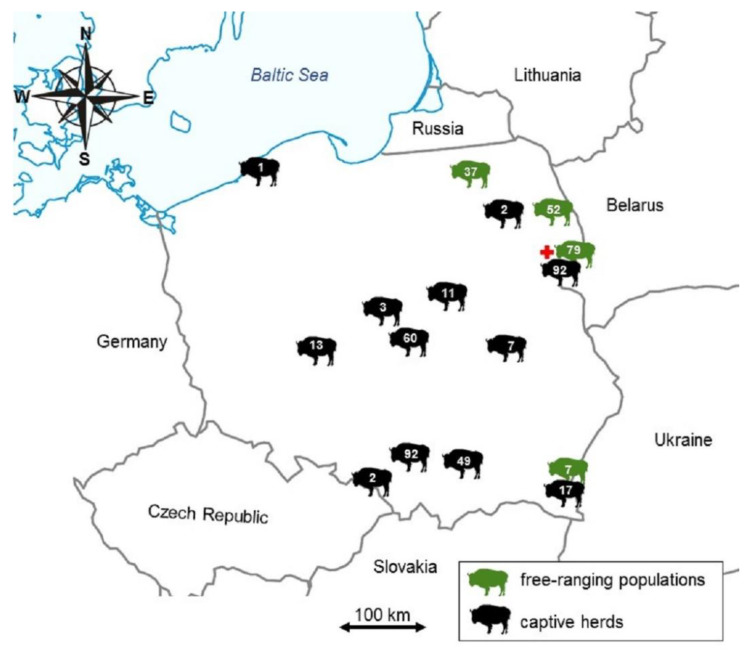
The map of Poland showing the numbers of European bison divided into free-ranging populations (green) and captive herds (black). The red cross indicates the location of single seropositive, 6-year-old male from wild population of Białowieska Forest.

A total of 523 serum samples were collected from European bison from 14 (out of 26 existing) different populations spread across the country including wild-ranging European bison from Białowieska (including Białowieża National Park) (*n* = 170), Borecka (*n* = 37) and Knyszyńska (*n* = 52) forests and several other herds kept in captivity between 2013 and 2018. The distribution and numbers of the studied European bison are presented in Figure 1. The samples originated both from female (*n* = 286) and male (*n* = 219) European bison aged between 5 days and 29 years. Samples were taken from pharmacologically immobilized (for placing collars with telemetric transmitters or diagnostic reasons), fallen or euthanized due to poor health individuals in accordance with the corresponding decisions of the Minister of the Environment and the General Director for Environmental Protection. The blood was collected immediately after immobilization or culling through the puncture of the external jugular vein (*vena iugularis externa*), less often from the tail vein (*vena caudalis mediana*). Blood from dead, necropsied animals was collected in the form of a clot from the heart or from body cavities. The decayed or extensively hemolyzed samples were excluded from the study to minimize the risk of false result. Blood was collected into the sterile 7–9 mL tubes, centrifuged within 24 h, and the obtained serum was frozen at −70 °C until analysis in the sample bank of the Department of Virology, NVRI, Poland.

### 2.2. ELISA (Enzyme-Linked Immunosorbent Assay)

Serum samples were tested for presence of antibodies to *Coxiella burnetii* using commercial ELISA test ID Screen Q Fever Indirect Multi-species (IDvet, Grabels, France). This kit is based on a mix of phase I and II antigens obtained after the purification and inactivation of a *C. burnetii* strain isolated from the placenta of an aborting cow. The test was performed according to the manufacturer’s instructions. Briefly, tested and control sera (negative control—NC and positive control—PC) were diluted 1:50 in Dilution Buffer 2 at the dilution plate and then, 100 μL of the diluted sera were transferred to the test plate. The plate was incubated for 45 ± 4 min at 21 °C (±5 °C) and washed 3 times with the washing solution. A conjugate working solution was prepared by diluting the concentrated Conjugate (10×) in Dilution Buffer 3 in a ratio of 1:10. An amount of 100 µL of conjugate working solution was added to all wells. The plates were incubated for 30 ± 3 min at 21 °C (±5 °C) and then washed 3 times with the washing fluid solution. An amount of 100 µL of Substrate solution was added to all wells and the plates were incubated in a dark place for 15 ± 2 min at 21 °C (±5 °C). The reaction was stopped by adding 100 µL Stop Solution to each well. The optical densities (OD) were read at 450 nm using an ELISA plate reader (Epoch, BioTek, Winooski, VT). The results were interpreted by calculation of the Sample to Positive percentage (S/P%) as $S/P\%=\frac{\left(ODsample-ODNC\right)}{\left(ODPC-ODNC\right)}\times 100$ The result was considered reliable if: the average OD_PC_ value is greater than 0.350 and the ratio of the average OD_PC_ value and the average OD_NC_ value is greater than 3. If S/P% ≤ 40%, the result was considered negative; if 40% < S/P% ≤ 50%, the result was considered doubtful; if S/P% > 50%, the result was considered positive.

### 2.3. Statistical Analysis

For statistical evaluation, STATA software version 11 (StataCorp., College Station, TX, USA) was used.

The percentage of *C. burnetii* seropositive animals and 95% confidence interval (CI) values were calculated using binomial exact test. The S/P values were analyzed by kernel estimated frequency plot. The univariate associations between the seropositivities to *C. burnetii*, environmental (origin, population type, health status) and individual-level (sex, age) variables were estimated using Fisher’s exact test.

## 3. Results

Only one (0.19%; 95% CI: 0.005–1.1) serum sample reacted positively in the ELISA. It derived form 6-year-old European bison bull (No 1983, born in 2007) culled due to poor body condition and severe balanoposititis from the free-living population in January 2013. Furthermore, two other samples were doubtful. These included also free-living males: a 3-month-old calf (No 2250; born in 2012) and 6-year-old bull (No 1984; born in 2007), which were also eliminated by culling from Białowieska forest in 2013. The results in relation to different variables are presented at Table 1.

**Figure 2:**
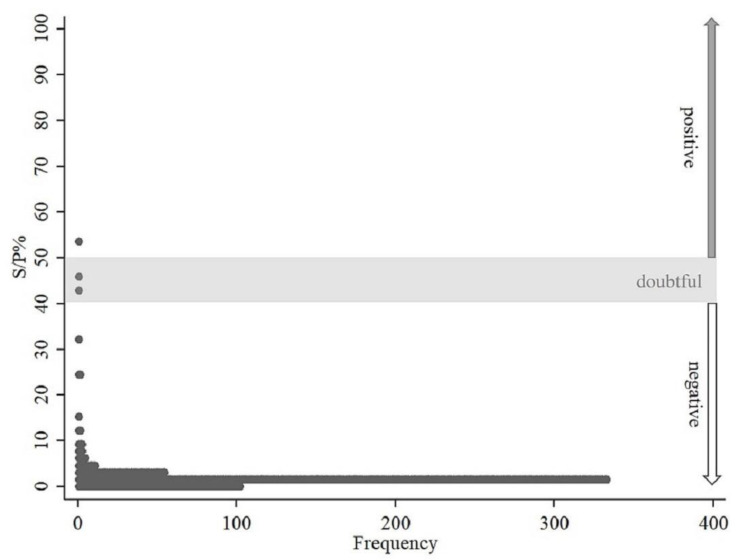
Dotblot distribution of Sample to Positive percentage (S/P%) values of ID Screen Q Fever Indirect Multi-species (IDvet, Grabels, France) testing for the presence of *Coxiella burnetii* antibodies in 523 sera of European bison (*Bison bonasus*) collected between 2013 and 2018 in 14 different locations in Poland.

The distribution of S/P% with a single peak skewed towards negative values may be observed in Figure 2.

## 4. Discussion

Nowadays, the increasing European bison population size and density, decreasing wild habitat and introduction strategies induces more frequent contact with other wild reservoirs and domestic animals at the shrinking interface. The population size of European bison in Poland and in Europe has tripled in the last twenty years [12]. Moreover, already three quarters of the animals are free ranging including in several new locations where the species was introduced or re-introduced after the extinction in Europe [12]. The role of the species as a reservoir of the zoonotic bacteria at present was therefore investigated using representative numbers of animals in the largest Polish populations as well as in the smaller, captive herds.

In our study, the only seropositive animal was a free-living bull. In 1980–1983 the studies of European bison culled at that time revealed antibodies to *C. burnetii* in a 2-year-old heifer. It was thought to be associated with the presence of a natural foci of Q fever in the Białowieska forest [9]. The outbreak was probably associated with the occurrence of Q fever epizootic outbreaks in cattle and sheep in neighboring villages [9]. However, despite continuous occurrence of Q fever in the domestic ruminants in 1980–1983, no transmission to European bison was observed. European bison males usually wander solitarily or in small male groups often approaching farmland and villages, where they can become into contact with domestic animals and humans. Meanwhile, females with calves and juveniles create so-called mixed groups, which rarely leave the forest, except for in winter, when they may come out onto fields or winter-feeding places in search for food. Therefore, it can be assumed that the exposure of *C. burnetii* in free-living bull in our study may have been associated with transmission of pathogen from domestic ruminants by sharing pastures. In the eighties, due to the occurrence of Q fever in domestic animals in north-eastern Poland, forty-seven free-ranging European bison from Borecka forest were examined for the presence of specific antibodies by the microagglutination test and complement fixation test (CFT). The high seroprevalence (76%) of *Coxiella burnetii* found in European bison suggested that they could be considered as a potential reservoir of Q fever [8]. Szarek et al. [15] have linked *C. burnetii* infection to the pathomorphological changes of heart and kidneys specific for Q fever observed in those studied European bison. Moreover, European bison–human transmission was suspected, since the infection was also confirmed in 10% of the employees of the Białowieża National Park (BNP) staff. However, further studies suggested that the Q fever foci were self-limited in the following years [10,11]. The results of our studies follow this trend.

The results of our studies, however, suggest that the exposure of European bison to *C. burnetii* is rather accidental if even occurring, which may be surprising, as the infections are quite common in cattle in the country [2]. Assuming that the positive result in only one free-living bull was not a false positive reaction, a transmission from cattle would be suspected. The seroprevalence of *C. burnetii* in cattle in the Podlaskie province, where two major European bison free-living populations of Białowieska and Knyszyńska forests are living reaches 48% [2]. The density of cattle in the region is also the highest in the country with the intensive milk production and increasing pasture grazing [16]. These findings suggest low susceptibility of European bison to *C. burnetii* infection despite the potential increasing risk with increasing contacts with domestic Q fever reservoirs in endemic areas.

The few reports on Q fever occurrence in wild ruminants in Europe suggest low seroprevalence of *C. burnetii*, and therefore their role in the transmission and maintaining the bacteria in the natural environment is rather doubtful. Low seroprevalences of *C. burnetii* were reported previously in wild ranging cervids and mouflon [6,7]. Higher seroprevalences were found in farmed deer, which may suggest increased exposure to *C. burnetii* connected to higher density of animals [17]. However, the factors modulating the risk of exposure to *C. burnetii* in wildlife are quite complex and should be evaluated individually [18]. Our study together with previous concerning *C. burnetii* infections in European bison [9,11] also suggest that they may be accidental hosts for the pathogen. Nevertheless, since *C. burnetii* may cause large reproductive loses to domestic ungulates, we should be cautious when it comes to endangered species as European bison and monitor the epidemic situation.

According to OIE [World Organisation for Animal Health; 19], at present, no gold standard technique is available in diagnosis of Q fever. Serological ELISA and direct detection and quantification by PCR should be considered as the methods of choice. Serological analyses may be carried out using ELISA, a complement fixation test (CFT) or indirect immunofluorescence assay (IFA). Among serological methods, ELISA is recommended for routine serological testing of animals for Q fever because it has a high sensitivity and a good specificity, and it is convenient for large-scale screening. The relative sensitivity is the lowest for CFT [19]. An antibody ELISA was used in our research. Some authors question the high test performance in other than small ruminants [20]; however, the assay was successfully used in Q fever epidemiological studies in other species [21]. In general, ELISA also shows improved sensitivity and specificity in relation to the complement fixation test [22,23], despite its performance possibly varying between different manufacturers, different antigens used for plate coating and the species to which it is applied [24]. The report on Q fever cases in European bison in the 1980s was based on CFT [8]; therefore, it could explain why further studies, including present, have differed on *C. burnetii* exposure in the species, despite potentially increasing risk. Studies on detecting antibodies against *C. burnetii* demonstrate only previous exposure to the pathogen, not current shedding of bacteria but they can be useful for epidemiological analysis of endemic areas [2,25]. Moreover, seropositivity without detection of pathogen in the sera may be indicative of chronic exposures to *C. burnetii* [26]. Moreover, some shedders of *C. burnetii* may be seronegative [27]. Therefore, some discrepancies in the results of serological versus PCR tests carried out on the same animals may be observed. Knap et al. [25] have detected the presence of specific antibodies in the sera of 60 out of 150 (40.4%) animals (cattle, sheep), while the presence of pathogen DNA was confirmed only in 14 out of 150 (9.3%) blood samples. Furthermore, Bellabidi et al. [21] found antibodies to *C. burnetii* in 75.54% of tested samples of camel sera and no DNA of the pathogen in sera of 138 seropositive animals. It is noteworthy that there are differences in the shedding of bacteria in different animal species. Cattle shed the bacteria almost exclusively in milk, goats mostly in milk with a minority shedding it in vaginal mucous or feces and sheep in feces, vaginal mucous and milk [27].

Since no tests dedicated to specific species, except for cattle, are recommended, and no tests are available for wildlife ruminants, the adaptation and optimization of the available test should be considered in further studies [5,18,23,24,28]. Since Q fever may be a potential emerging disease in the areas where contacts between wildlife and humans or domestic animals increase, the need for a reliable test for monitoring of wildlife is growing. Frosinski et al. [29] have also discussed the need for cut-off adaptation, which may be beneficial to obtaining good quality prevalence data. In our study, some samples gave slightly higher S/P% value approaching the cut-off value for doubtful results. However, optimizing cut-off value would require testing a higher number of known *C. burnetii* seropositive samples collected from the species which are not available at the moment (Figure 2). Further studies are needed.

## 5. Conclusions

The only seropositive European bison found in this study was a free-living bull which suggests possible transmission from domestic cattle by sharing pastures; however, a false positive result may be also suspected. The transmission of *C. burnetii* into the European bison was rather accidental in the country and its role as an important wild reservoir is questionable. Since no tests are available for wildlife ruminants there is a need for the adaptation of the available tests in order to monitor possible *C. burnetii* circulation in the sylvatic environment.

## Figures and Tables

**Table 1:** Descriptive statistics of seropositive to *Coxiella burnetii* European bison (*Bison bonasus*) with regard to their different characteristics.

	Number Positive/ Examined	% (95% Confidence Interval)
Location (Global Positioning System) (N * = 523)		
Bałtów (51°1′3.759″ N 21° 32′30.098″ E)	0/7	0 (0–41.0)
Białowieska forest (52°42′9.861″ N 23°51′4.52″ E)	1/171 **	0.58 (0.1–3.2)
Bieszczady mountains (49°7′12.898″ N 22°45′0.782″ E)	0/24	0 (0–14.2)
Borecka forest (54°5′18.09″ N 21°55′21.467″ E)	0/37	0 (0–9.5)
Gołuchów (51°50′58.047″ N 17°55′50.863″ E)	0/12	0 (0–26.5)
Kiermusy (53°12′7.699″ N 22°42′44.245″ E)	0/2	0 (0–84.2)
Knyszyńska forest (53°15′35.036″ N 23°38′37.11″ E)	0/52	0 (0–6.8)
Niepołomice (50°1′45.67″ N 20°20′46.365″ E)	0/49	0 (0–7.2)
Pszczyna (49°58′29.284″ N 18°55′52.465″ E)	0/92	0 (0–3.9)
Smardzewice (51°28′39.975″ N 20°3′0.964″ E)	0/60	0 (0–6.0)
Strzelinko (54°31′45.236″ N 16°56′58.023″ E)	0/1	0 (0–97.5)
Ustroń (49°42′58.955″ N 18°49′59.765″ E)	0/2	0 (0–84.2)
ZOO Łódź (51°45′39.629″ N 19°24′45.333″ E)	0/3	0 (0–70.8)
ZOO Warsaw (52°15′28.9″ N 21°1′21.035″ E)	0/11	0 (0–28.5)
**Population type (*N* = 523)**		
free-living	1/179 **	0.56 (0.1–3.1)
captive	0/344	0 (0–1.1)
**Gender (*N* = 505)**		
female	0/281	0 (0–1.3)
male	1/224 **	0.44 (0.01–2.4)
**Age group (*N* = 474)**		
≤1 year old	0/98 ***	0 (0–3.7)
2–3 years old	0/114	0 (0 = 3.2)
≥4 years old	1/262 ***	0.38 (0.1–2.1)
**Health status (*N* = 501)**		
immobilized (apparently healthy)	0/348	0 (0–1.1)
eliminated (by culling)	1/134 **	0.74 (0.02–4.1)
fallen	0/15	0 (0–21.8)
traffic accident	0/4	0 (0–60.2)

* number of examined in the category (taking into account the missing data); ** two and *** one additional doubtful result was detected.

## Data Availability

Raw data are available upon request from the corresponding author.
